# Supplementary material for: TAD boundary deletion causes PITX2-related cardiac electrical and structural defects
Source: Nat Commun. 2024 Apr 20;15:3380. doi: 10.1038/s41467-024-47739-x (PMC11032321; doi:10.1038/s41467-024-47739-x)
Supplement: Supplementary file 1 — Supplementary Information [file 41467_2024_47739_MOESM1_ESM.pdf]

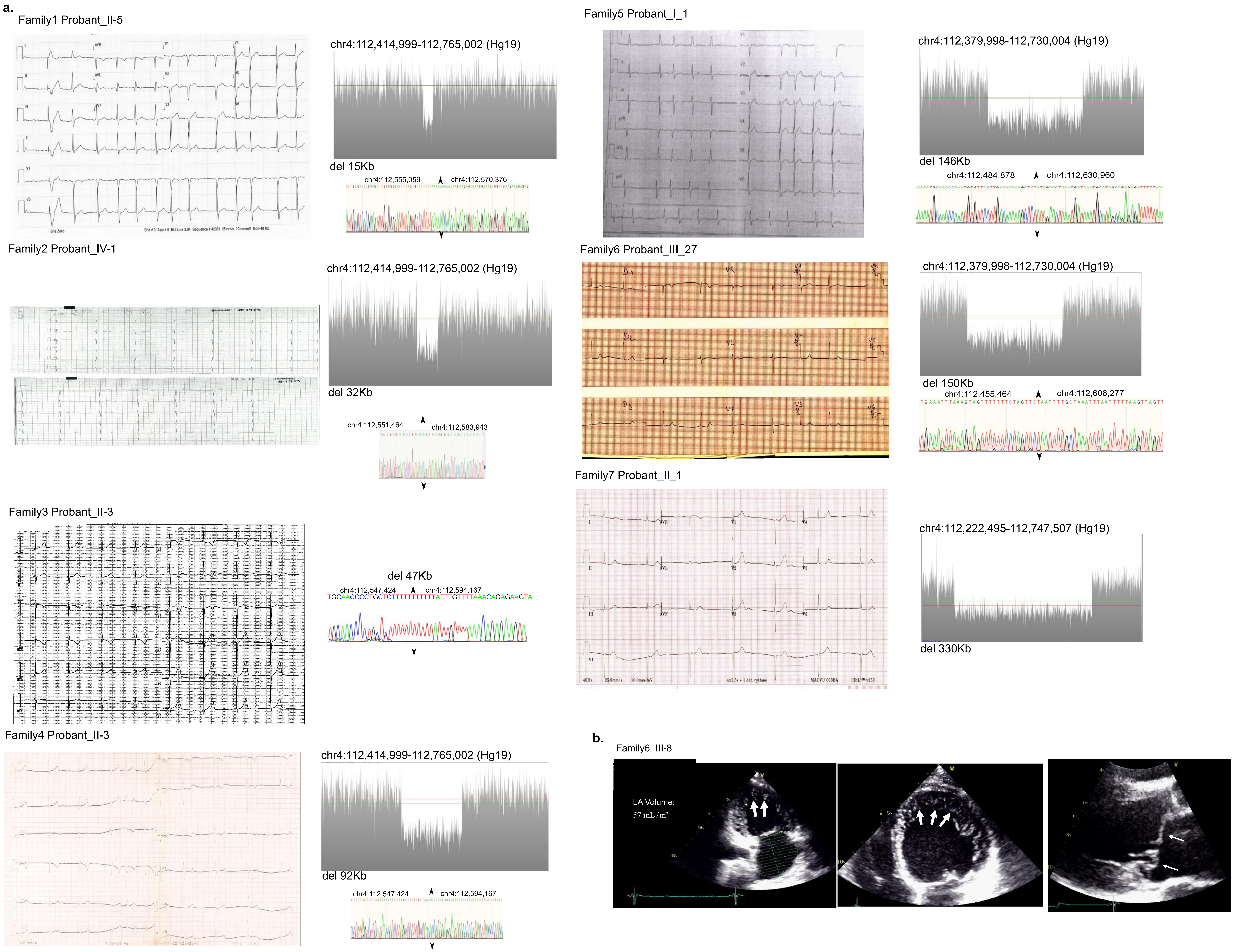
a.
Family1 Probant_II-5
chr4:112,414,999-112,765,002 (Hg19)
del 15Kb
chr4:112,555,059
chr4:112,570,376
Family2 Probant_IV-1
chr4:112,414,999-112,765,002 (Hg19)
del 32Kb
chr4:112,551,464
chr4:112,583,943
Family3 Probant_II-3
del 47Kb
chr4:112,547,424
chr4:112,594,167
Family4 Probant_II-3
chr4:112,414,999-112,765,002 (Hg19)
del 92Kb
chr4:112,547,424
chr4:112,594,167
Family5 Probant_I_1
chr4:112,379,998-112,730,004 (Hg19)
del 146Kb
chr4:112,484,878
chr4:112,630,960
Family6 Probant_III_27
chr4:112,379,998-112,730,004 (Hg19)
del 150Kb
chr4:112,455,464
chr4:112,606,277
Family7 Probant_II_1
chr4:112,222,495-112,747,507 (Hg19)
del 330Kb
b.
Family6_III-8
LA Volume:
57 mL /m²

Supplementary Fig.1: **Electrocardiogram and molecular features of probands and echocardiographic phenotype of individual III-8 Familiy#6.**

a, Surface 12-lead ECG of the proband in the 7 families. WGS and/or Electropherogram highlighting the deletion breaking point in 6 families. The deletion is highlight by reduction in sequencing density visualize by SwingBamCov tool of Jvarkit. **b,** Echocardiographic phenotype of the individual III-8 Family#6 presenting sinus node dysfunction and atrial fibrillation, associates left ventricular myocardial non-compaction (thick white arrows) and left atrial dilatation (57 mL/m²) on the left and middle images. Moderate mitral valve prolapse (thin white arrow) on the right image.

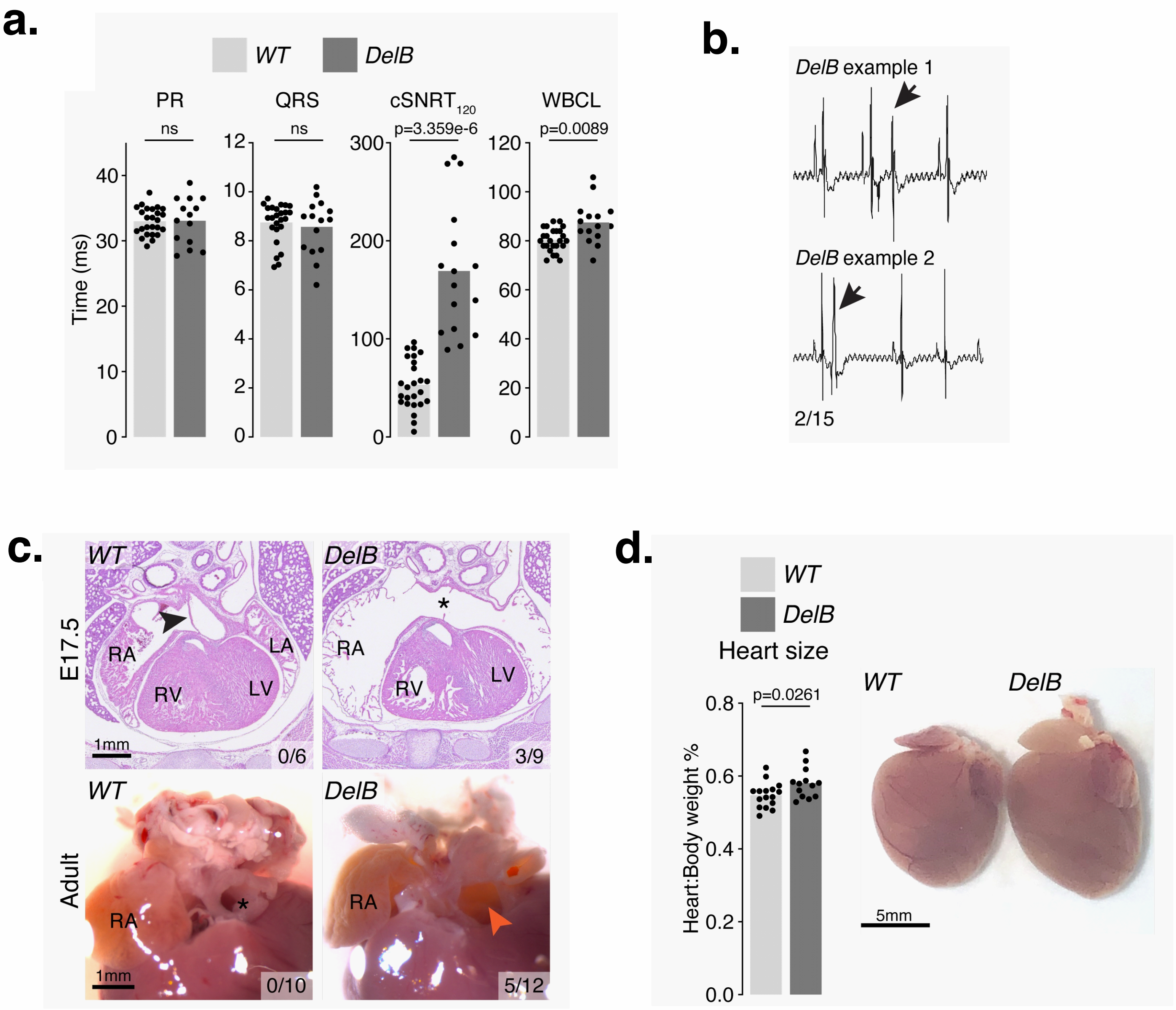
a.
WT
DelB
PR
QRS
cSNRT120
WBCL
ns
ns
p=3.359e-6
p=0.0089
Time (ms)
0
10
20
30
40
0
2
4
6
8
10
12
0
100
200
300
0
20
40
60
80
100
120
b.
DelB example 1
DelB example 2
2/15
c.
WT
DelB
E17.5
RA
LA
RV
LV
*
1mm
0/6
3/9
Adult
RA
0/10
5/12
d.
WT
DelB
Heart size
p=0.0261
Heart:Body weight %
0.0
0.2
0.4
0.6
0.8
5mm

Supplementary Fig. 2: ***DelB*** **mice show in vivo conduction and morphological abnormalities**. **a,** Graphs show individual and average ECG and pacing measurement for PR and QRS interval, heart rate-corrected sinus node recovery times (cSNRT) at 120ms and Wenckebach cycle leght (WBCL) of control (n=25)and *DelB* (n=15) mice. Significance for each parameter was determined with Welch's t test (PR, sSNRT, WBCL) and Mann-Whitney test (QRS duration). **b,** ECG traces show two examples of *DelB* individuals showing escaped beats (arrows) (WT, 0/25, *DelB* 2/15). **c,** matched histological sections of E17.5 WT and *DelB* fetuses showing atrial septation (black arrowhead), or incomplete atrial septation (astrisk). Ratios in lower right corner of each image indicate affected individuals (p=0.0278). Representative image of WT and *DelB* isolated adult heart in which the left atrium was removed to observe dye in the right leakage into the left atrium from injection of dye in the right atrium (orange arrowhead); Ratios in the lower right corner of each image indicate individuals in which dye was found in the left atrium (p=0.0396). Statistical significance was determined with Fisher's exact test (fetal and adult atrial defects). RA, right atrium; RV, right ventricle; LV, left ventricle; LA, left atrium. **d,** Graph denotes individual and average percentage heart to body weight rations of adult WT (n=16) and *DelB* (n=13) mice. Representative image of WT and *DelB* isolated hearts, side by side. Source data are provided as a Source Data file.

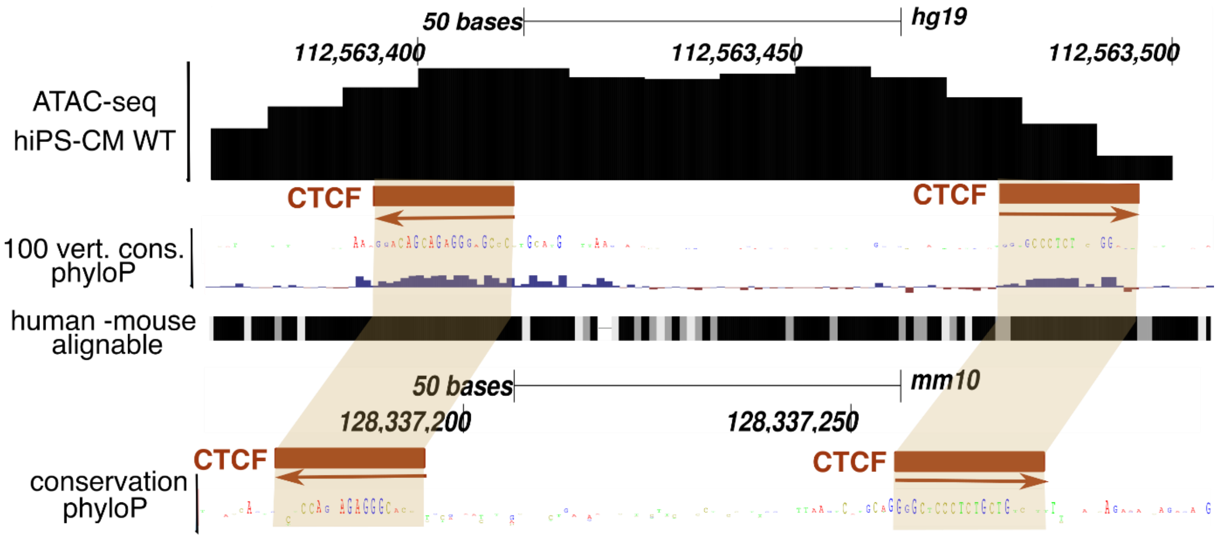

50 bases
hg19
112,563,400
112,563,450
112,563,500
ATAC-seq
hiPS-CM WT
CTCF
CTCF
100 vert. cons.
phyloP
human -mouse
alignable
50 bases
mm10
128,337,200
128,337,250
CTCF
CTCF
conservation
phyloP

Supplementary Fig. 3: screenshot of the UCSC browser representing the CTCF binding sites sequences orientation at the boundary of the PITX2-TAD and the non-coding TAD. Top panel: human genome *Hg19*, bottom panel: mouse genome *mm10*.

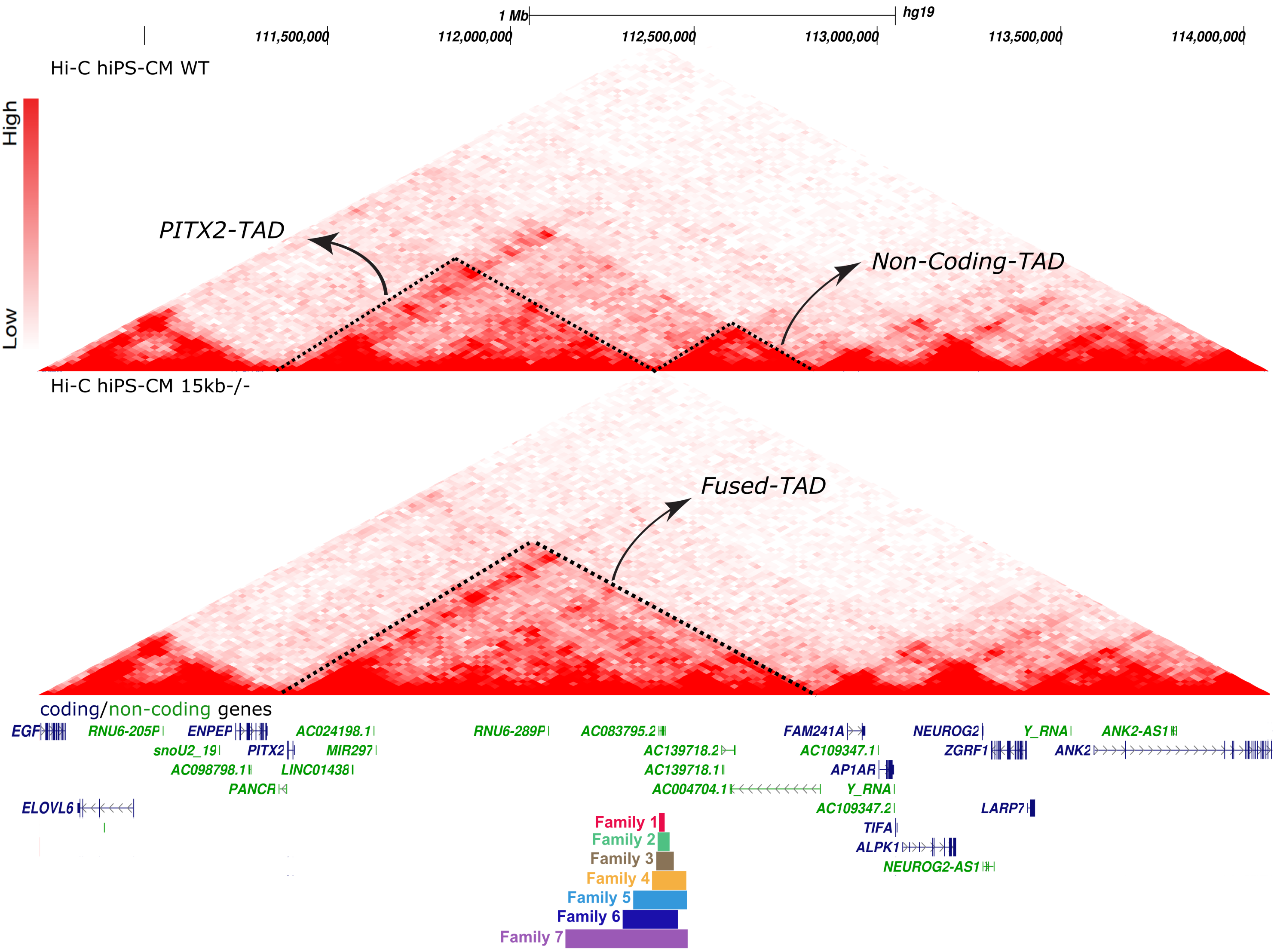
1 Mb
hg19
111,500,000
112,000,000
112,500,000
113,000,000
113,500,000
114,000,000
Hi-C hiPS-CM WT
High
Low
PITX2-TAD
Non-Coding-TAD
Hi-C hiPS-CM 15kb-/-
Fused-TAD
coding/non-coding genes
EGF
RNU6-205P
ENPEP
AC024198.1
RNU6-289P
AC083795.2
FAM241A
NEUROG2
Y_RNA
ANK2-AS1
snoU2_19
PITX2
MIR297
AC139718.2
AC109347.1
ZGRF1
ANK2
AC098798.1
LINC01438
AC139718.1
AP1AR
PANCR
AC004704.1
Y_RNA
ELOVL6
AC109347.2
LARP7
TIFA
ALPK1
NEUROG2-AS1
Family 1
Family 2
Family 3
Family 4
Family 5
Family 6
Family 7

Supplementary Fig. 4: 3D chromatin remodeling induces the fusion of the PITX2 TAD and non-coding TAD. Annotation of the 4q25 region with Hi-C map interaction profile from hiPSC-CM WT and hiPSC-CM 15Kb-/- in UCSC Genome browser. Dashed triangles delimit the PITX2-TAD, Non-coding-TAD, and fused TAD. 7 Deletions sizes are represented in different horizontal color lines: red/Family#1 (chr4:112555060-112570371), green/Family#2, brown/Family#3, yellow/Family#4, Blue/Family#5, dark blue/Family#6 and purple/Family#7

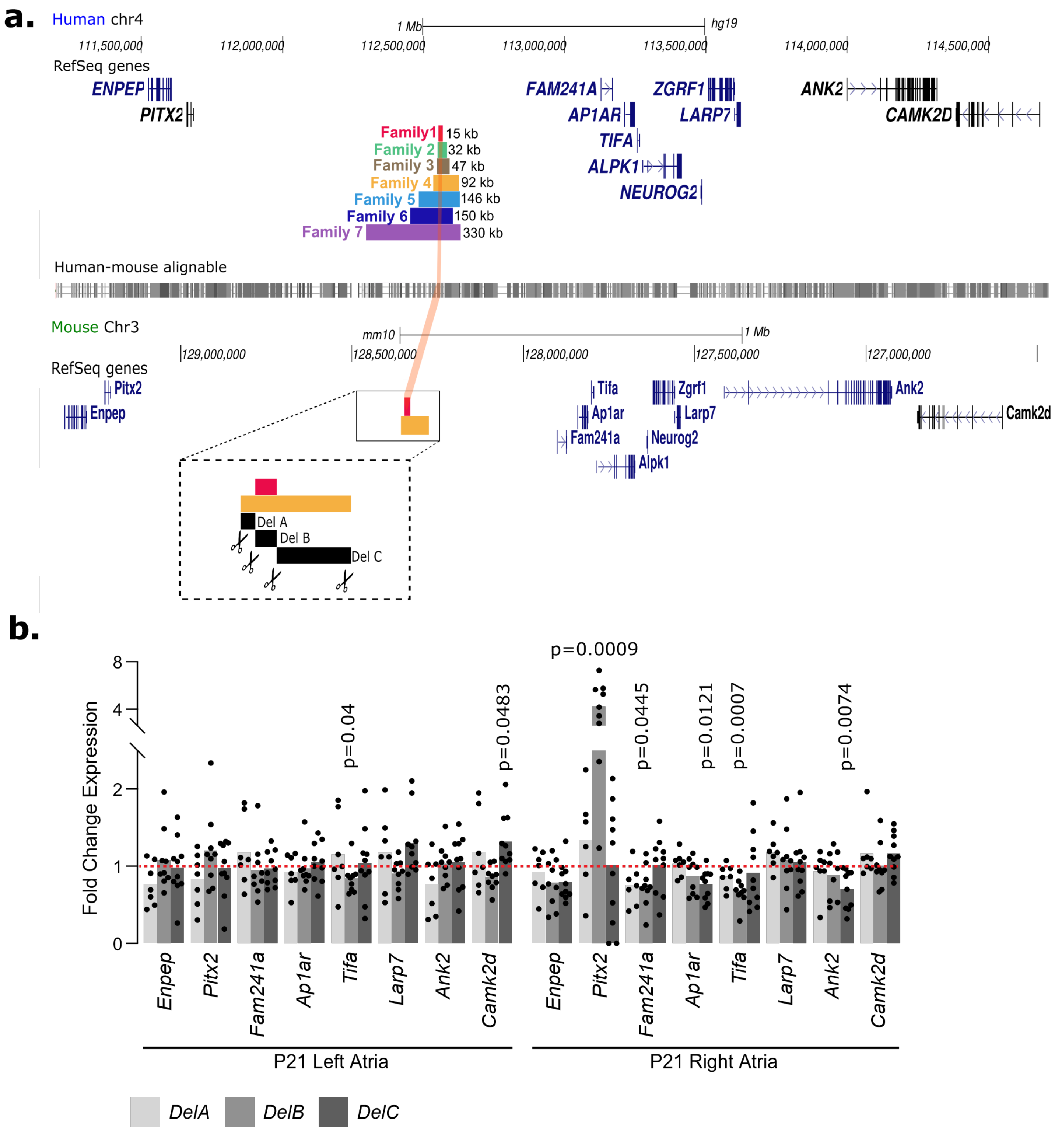

a.
Human chr4
1 Mb
hg19
111,500,000
112,000,000
112,500,000
113,000,000
113,500,000
114,000,000
114,500,000
RefSeq genes
ENPEP
PITX2
FAM241A
AP1AR
TIFA
ALPK1
NEUROG2
ZGRF1
LARP7
ANK2
CAMK2D
Family1 15 kb
Family 2 32 kb
Family 3 47 kb
Family 4 92 kb
Family 5 146 kb
Family 6 150 kb
Family 7 330 kb
Human-mouse alignable
Mouse Chr3
mm10
1 Mb
129,000,000
128,500,000
128,000,000
127,500,000
127,000,000
RefSeq genes
Pitx2
Enpep
Tifa
Ap1ar
Fam241a
Alpk1
Zgrf1
Larp7
Neurog2
Ank2
Camk2d
Del A
Del B
Del C
b.
Fold Change Expression
8
4
2
1
0
p=0.04
p=0.0483
p=0.0009
p=0.0445
p=0.0121
p=0.0007
p=0.0074
Enpep
Pitx2
Fam241a
Ap1ar
Tifa
Larp7
Ank2
Camk2d
P21 Left Atria
P21 Right Atria
DelA
DelB
DelC

Supplementary Fig. 5: Expression of the surrounding gene deletion in WT and Fold change expression level of these genes in DelA and DelC vs WT. a, (Top) Overview of the 4q25 human loci. Genes are represented in black and blue. 7 Deletions sizes are represented in different horizontal color lines: red/Family#1 (chr4:112555060-112570371), green/Family#2, brown/Family#3, yellow/Family#4, Blue/Family#5, dark blue/Family#6 and purple/Family#7 in UCSC Genome browser. Human-mouse conservation is measured by Cons 46-way (phastCons and phyloP score) (Bottom) representation of the reverse orthologous mouse locus provided by USCS Genome browser. Genes are represented in black and blue. Homozygous deletions generated in the mouse model are represented in black horizontal lines. DelA and DelC correspond to the regions between orthologous deletion of Family#1 and Family#4, *DelB* corresponding to the orthologous deletion of Family#1. **b,** Fold change expression levels of detectable TAD genes in P21 DelA (light grey, n=7), DelC (medium grey, n=9), and DelC (dark grey, n=10) vs control littermates (dotted red line) in left and right atria. Statistically significant changes were determined across four genotypes and within each tissue type using Kruskal-Wallis test followed by pairwise comparisons with Dunn's multiple comparison tests. Significant p values adjusted for multiple testing shown within graph. Source data are provided as a Source Data file.

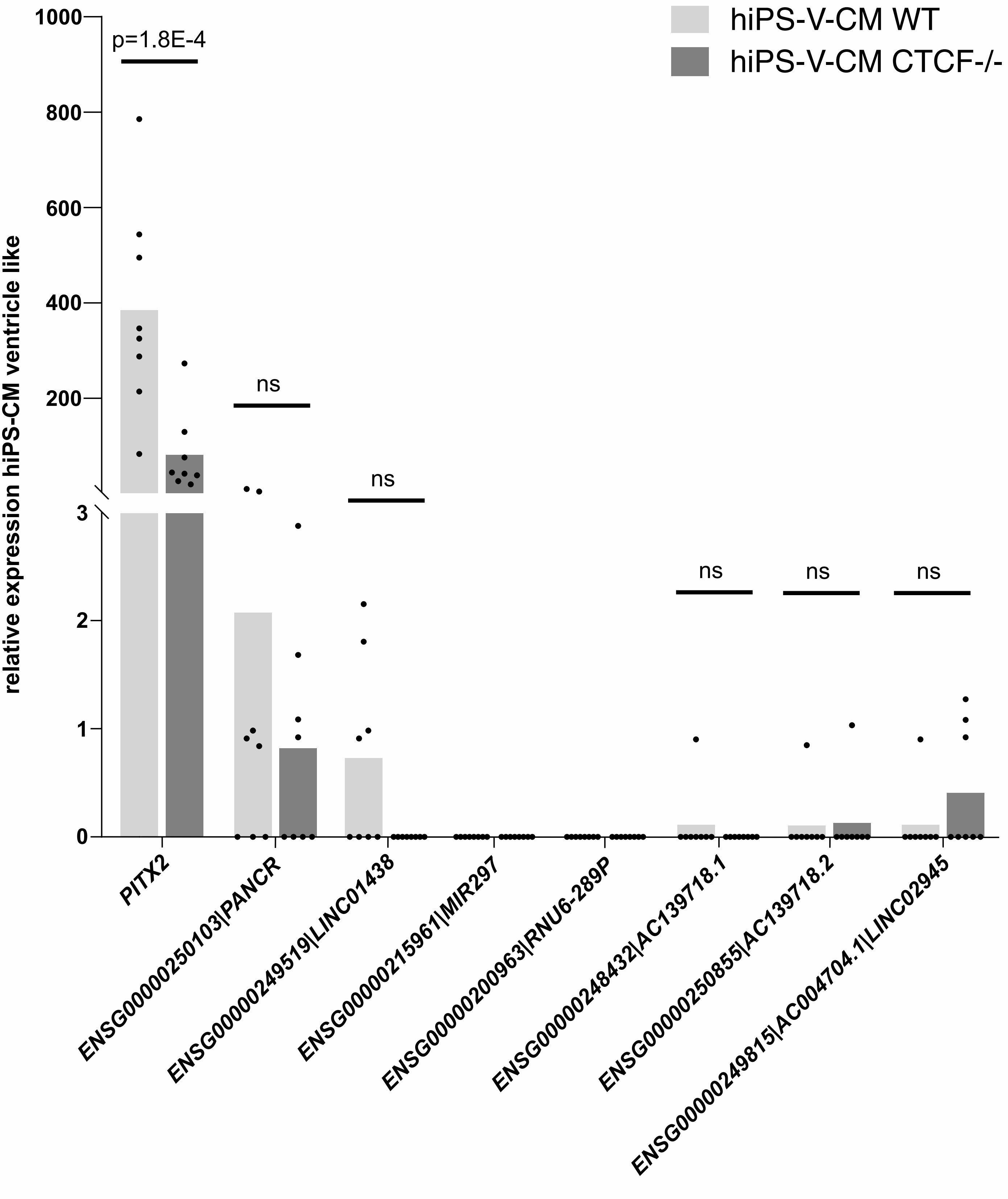

hiPS-V-CM WT
hiPS-V-CM CTCF-/-
relative expression hiPS-CM ventricle like
1000
800
600
400
200
3
2
1
0
p=1.8E-4
ns
ns
ns
ns
ns
PITX2
ENSG00000250103|PANCR
ENSG00000249519|LINC01438
ENSG00000215961|MIR297
ENSG00000200963|RNU6-289P
ENSG00000248432|AC139718.1
ENSG00000250855|AC139718.2
ENSG00000249815|AC004704.1|LINC02945

Supplementary Fig. 6: Expression of the LncRNA present in the PITX2 TAD and the non-coding TAD in hiPSC-VCM WT and hiPSC-VCM CTCF-/- ventricle like. Relative expression RNA-seq of PANCR, LINC01438, MIR297 and RNI6-289P present in the PITX2-TAD and AC139718.1, AC139718.2 and LINC02945 present in the non-coding TAD normalized with deseq2 normalization in hiPSC-V-CM WT and CTCF -/- (n=8, n=8). Source data are provided as a Source Data file.
