# Supplementary material for: TAD boundary deletion causes PITX2-related cardiac electrical and structural defects
Source: Nat Commun. 2024 Apr 20;15:3380. doi: 10.1038/s41467-024-47739-x (PMC11032321; doi:10.1038/s41467-024-47739-x)
Supplement: Supplementary file 3 — Description of Additional Supplementary Files [file 41467_2024_47739_MOESM3_ESM.pdf]

Description of Additional Supplementary Files

Supplementary Data 1: Individual clinical data carrying or not the deletion

Supplementary Data 2: Whole tissue RNA-seq of WT and DelB SAN tissue

Supplementary Data 3: GO-term analysis using PANTHER of upregulated genes in whole-tissue RNA-seq of the DelB SAN

Supplementary Data 4: GO-term analysis using PANTHER of downregulated genes in whole-tissue RNA-seq of the DelB SAN

Supplementary Data 5: Comparison of genes differentially expressed in the DelB SAN and genes enriched or depleted in PCM

Supplementary Data 6: GO-term analysis using PANTHER of upregulated genes in whole-tissue RNA-seq of the DelB SAN that are PCM enriched

Supplementary Data 7: GO-term analysis using PANTHER of downregulated genes in whole-tissue RNA-seq of the DelB SAN that are PCM enriched

Supplementary Data 8: GO-term analysis using PANTHER of downregulated genes in whole-tissue RNA-seq of the DelB SAN that are PCM depleted

Supplementary Data 9: GO-term analysis using PANTHER of upregulated genes in whole-tissue RNA-seq of the DelB SAN that are PCM depleted

Supplementary Data 10: Prevalence of structural variant overlapping CTCF binding site in patients presenting lone electrical defect

Supplementary Data 11: qPCR primers for deletion interrogation

Supplementary Data 12: Primer for deletion breaking point sequencing

Supplementary Data 13: gRNA for CRISPR to generate mutant mice

Supplementary Data 14: Primer for genotyping mutant mice

Supplementary Data 15: Primers for quantitative real-time PCR on mouse model

Supplementary Data 16 gRNA for CRISPR to generate isogenic model on iPS cells

Supplementary Data 17: Primer for genotyping isogenic models

Supplementary Data 18: Primers for quantitative real-time PCR on isogenic hiPS-CM model
